# Relationship between Levels of Brain-Derived Neurotrophic Factor and Metabolic Parameters in Patients with Type 2 Diabetes Mellitus

**DOI:** 10.1155/2014/978143

**Published:** 2014-12-22

**Authors:** Banu Boyuk, Serife Degirmencioglu, Hande Atalay, Savas Guzel, Ayse Acar, Aslan Celebi, Ismail Ekizoglu, Caglar Simsek

**Affiliations:** ^1^Department of Internal Medicine, Gaziosmanpasa Taksim Education and Research Hospital, Siraselviler Caddesi No. 112, 34433 Istanbul, Turkey; ^2^Department of Biochemistry, Namik Kemal University, Turkey; ^3^Department of Diabetic Patient Education and Care Policlinic, Gaziosmanpasa Taksim Education and Research Hospital, Turkey

## Abstract

*Background and Aim*. Studies have suggested that brain-derived neurotrophic factor (BDNF) plays a role in glucose and lipid metabolism and inflammation. The aim of this study was to evaluate the relationship between serum BDNF levels and various metabolic parameters and inflammatory markers in patients with type 2 diabetes mellitus (T2DM). *Materials and Methods*. The study included 88 T2DM patients and 33 healthy controls. Fasting blood samples were obtained from the patients and the control group. The serum levels of BDNF were measured with an ELISA kit. The current paper introduces a receiver-operating characteristic (ROC) generalization curve to identify cut-off for the BDNF values in type 2 diabetes patients. *Results*. The serum levels of BDNF were significantly higher in T2DM patients than in the healthy controls (206.81 ± 107.32 pg/mL versus 130.84 ± 59.81 pg/mL; *P* < 0.001). They showed a positive correlation with the homeostasis model assessment of insulin resistance (HOMA-IR) (*r* = 0.28; *P* < 0.05), the triglyceride level (*r* = 0.265; *P* < 0.05), and white blood cell (WBC) count (*r* = 0.35; *P* < 0.001). In logistic regression analysis, age (*P* < 0.05), body mass index (BMI) (*P* < 0.05), C-reactive protein (CRP) (*P* < 0.05), and BDNF (*P* < 0.01) were independently associated with T2DM. In ROC curve analysis, BDNF cut-off was 137. *Conclusion*. The serum BDNF level was higher in patients with T2DM. The BDNF had a cut-off value of 137. The findings suggest that BDNF may contribute to glucose and lipid metabolism and inflammation.

## 1. Introduction

Brain-derived neurotrophic factor (BDNF) is a member of the neurotrophin family, which includes nerve growth factor, neurotrophin-3, and neurotrophin-4/5 [1–4]. The role of BDNF in cell differentiation, neural growth, synaptic connectivity, and maintenance of target neurons is well established; it has also been implicated in synaptic plasticity of brain function, such as learning and memory [5, 6].

In addition to the role of BDNF in neurological disorders, recent studies reported that peripheral injection of BDNF exerts hypophagic and hypoglycemic effects on obese hyperglycemic animals but not on normal animals, pointing to antiobesity and antidiabetic effects [7–10]. BDNF mutant mice developed mature onset obesity, characterized by an increase in body weight [11]. In the same study, the mutant mice displayed elevated serum leptin, insulin, glucose, and cholesterol. Data from animal experiments and human studies suggested that BDNF may contribute to glucose metabolism and have a pathogenic role in the development of type 2 diabetes mellitus (T2DM) in humans [12]. According to recent studies, BDNF is associated with systemic inflammatory conditions, such as diabetes, acute coronary syndrome, and atherosclerosis [13, 14]. The aim of this study was to evaluate the relationship between serum BDNF levels and various metabolic parameters and inflammatory markers in patients with T2DM.

## 2. Materials and Methods

### 2.1. Study Population

This study was performed at the GOP Taksim Education and Research Hospital outpatient department of internal medicine. It included 88 patients (38 males and 50 females) and 33 control subjects (17 males and 16 females). The presence of diabetes was based on a previous diagnosis of T2DM or a random plasma glucose level of 200 mg/dL or higher, together with classical features of DM, such as polyuria, polydipsia, polyphagia, and weight loss, or a fasting blood glucose level of >126 mg/dL or higher or a HbA1C level of 6.5% or higher. Exclusion criteria were the presence of systemic diseases: neoplastic, inflammatory, and infectious diseases. The study protocol was approved by GOP Taksim Research and Education Hospital ethics committee, Istanbul. Informed written consent was obtained from all the participants (patients and controls) after receiving a full explanation about the study and its purpose.

### 2.2. Measurements

Hypertension was defined as antihypertensive drug use or systolic blood pressure ≥140 mmHg and/or diastolic blood pressure ≥90 mmHg. Body mass index (BMI) was obtained using the formula weight (kg)/height (m)^2^. Obesity was defined as a BMI >30 kg/m^2^. Waist circumference was measured in a standing position at the level of the umbilicus.

Blood samples were obtained after overnight fasting. Serum cholesterol, triglyceride, and high-density lipoprotein cholesterol (HDL-C) were measured by enzymatic colorimetric methods with commercially available kits (COBAS 311, Roche Diagnostics GmbH, Mannheim, Germany), and low-density lipoprotein cholesterol C (LDL-C) was calculated according to the Friedewald formula. Serum glucose measures were determined enzymatically using the hexokinase method (Roche Diagnostics GmbH, Mannheim, Germany). Blood HbA1c was determined with a COBAS 311 analyzer using the particle-enhanced immunoturbidimetric method (Roche Diagnostics, Mannheim, Germany). Final results were expressed as percent HbA1c of the total Hb according to the protocol of the Diabetes Control and Complications Trial/National Glycohemoglobin Standardization Program (DCCT/NGSP). The particle-enhanced immunoturbidimetric method with a Behring Nephelometer BN-100 (Behring Diagnostic, Frankfurt, Germany) was used to measure C-reactive protein (CRP). The sensitivity of the test was 0.1 mg/L. White blood cell (WBC) levels were measured with an automatic hematology analyzer (Beckman Coulter, Brea, CA, USA). The erythrocyte sedimentation rate (ESR) was determined with the Westergren method using an established normal range of 0–20 mm/1 hr. Ferritin was measured with an electrochemiluminescence immunoassay (Roche Hitachi Modular E 170). The insulin level was determined with an electrochemiluminescence immunoassay (Roche Diagnostics, Mannheim, Germany) on an automated Roche Cobas E 411 (Roche Diagnostics). The homeostasis model assessment of insulin resistance (HOMA-IR) index was calculated from the fasting blood glucose and fasting serum insulin concentrations by the formula: HOMA-IR = fasting serum insulin (*μ*U/mL) × fasting blood glucose (mmol/L)/22.5. Serum BDNF was quantified using an ELISA (ChemiKine BDNF Sandwich ELISA kit, CYT306; Millipore Bioscience Research Reagents; USA and Canada) following the instructions of the manufacturer. The intra-assay and interassay coefficients of variation were 3.7 and 8.5%, respectively.

### 2.3. Statistical Analyses

Number Cruncher Statistical System (NCSS) 2007 and Power Analysis and Sample Size (PASS) 2008 Statistical Software (Utah, USA) programs were used for the statistical analysis. Descriptive statistical methods (mean, standard deviation, frequency, ratio, minimum, and maximum) were used to evaluate the study data. Student's *t*-test was used for normally distributed quantitative parameters. Mann-Whitney *U* test was used for quantitative parameters that were not normally distributed. Yates continuity correction test was used for the comparison of the qualitative parameters. Spearman's correlation coefficients were employed for bivariate associations of BDNF and other covariates. Values of *P* < 0.05, *P* < 0.01, and *P* < 0.001 were accepted as statistically significant.

## 3. Results

### 3.1. Clinical Characteristics of the Study Subjects

The clinical characteristics of the study subjects are shown in Table 1. There were no significant differences in the age and gender or triglyceride, total cholesterol, HDL, LDL, WBC, and ferritin levels between the T2DM patients and control subjects. The T2DM patients had a significantly higher BMI, waist circumference, systolic pressure, diastolic pressure, fasting plasma glucose, fasting insulin, HOMA-IR, HbA1C, ESR, and CRP levels than the control subjects (*P* < 0.001). Among the patients, 64% were using oral antidiabetic drug only, 18% were using oral antidiabetic drug plus insulin, and 18% were using insulin only. On the whole, 30% of patients were taking lipid lowering drug, 61% of patients were taking antihypertension drug, and 9% were taking none. Mean diabetes duration for enrolled subjects is 4.03 ± 3.38 (not shown in table).

35.2% of patients have cigarette smoking history (Table 1). Control group was composed of nonsmokers. Thus, smoking may have role in high serum BDNF levels of patient group.

### 3.2. Relationships between Serum BDNF Level and Other Variables

The serum BDNF levels showed a positive correlation with HOMA-IR (*r* = 0.28; *P* < 0.05), the triglyceride level (*r* = 0.265; *P* < 0.05), and the WBC level (*r* = 0.35; *P* < 0.001) in T2DM as shown in Table 2.

In the logistic regression analysis, age (*P* < 0.05), BMI (*P* < 0.05), CRP (*P* < 0.05), and BDNF (*P* < 0.001) were independently associated with T2DM (Table 3).

### 3.3. Serum BDNF Levels of T2DM Patients

**Figure 1:**
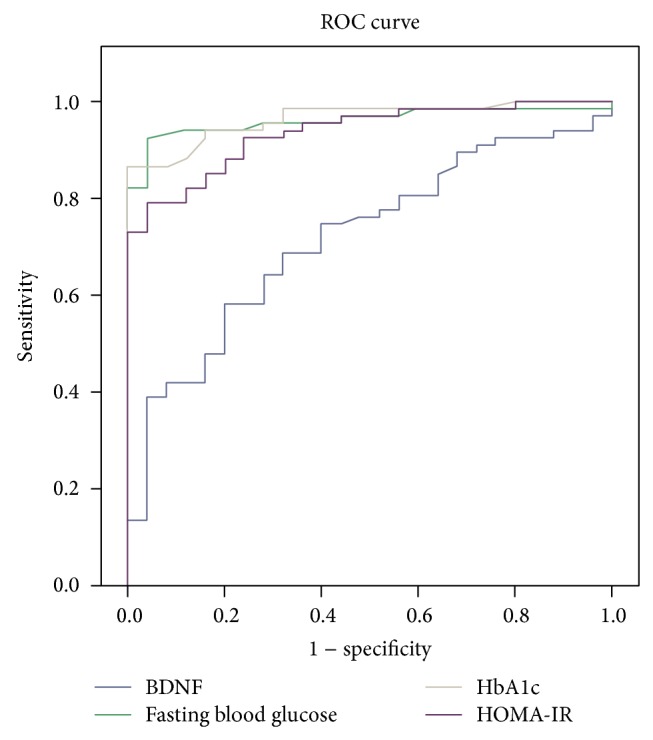
ROC curve.

The serum BDNF levels were significantly higher in the T2DM patients compared to the healthy controls (206.81 ± 107.32 pg/mL versus 130.84 ± 59.81 pg/mL, *P* < 0.001). A receiver operating characteristic curve (ROC) analysis and diagnostic screening tests were used to determine the cut-off point for BDNF (Table 4). Patients, who had a BDNF level more than 137 pg/mL, were catching study group levels with a sensitivity of 71.79%, a specificity of 68%, a positive predictive value of 87.5%, and a negative predictive value of 43.59%. The area under the ROC and the standard deviation were 71.8% and 5.6%, respectively. HbA1c was the best predictor, followed by fasting blood glucose and HOMA-IR (Figure 1).

## 4. Discussion

In this study, we investigated changes in the plasma BDNF level and correlations between BDNF and clinical and biochemical parameters in T2DM patients. We found that the serum BDNF of T2DM patients was significantly higher than that of control subjects. A previous study showed that serum BDNF levels were elevated in newly diagnosed female T2DM patients compared to healthy subjects [15]. Similarly, a recent study reported that serum BDNF was significantly elevated in T2DM patients compared to healthy controls [16]. There are various potential mechanisms that link BDNF and development of type 2 diabetes. In animal experiments, it is shown that, by suppressing PPAR-alpha and fibroblast growth factor 21, BDNF might facilitate insulin resistance and dyslipidemia and thus has antidiabetic and lipid lowering effects [17]. Furthermore, some researchers claim that BDNF treatment reported to lower blood glucose in diabetic models [18]. Similarly, Yamanaka et al. demonstrated that treatment with BDNF prevents age-related increase in blood glucose and development of diabetes in prediabetic db/db mice [8]. Together with the data derived from animal experiments, authors suggest that exogenous BDNF administration shows its antidiabetic and antilipidemic effects similar to thiazolidinediones [19]. It is demonstrated that Taşçi et al. speculate that compensatory increase in BDNF synthesis may occur in untreated hyperglycemia [20]. However, the diabetic patients in our study were not newly diagnosed; thus, the duration of diabetes alone could not explain the elevated level of serum BDNF.

On the other hand, Fujinami et al. reported that serum BDNF levels were significantly lower in patients with advanced T2DM compared to control subjects [12]. Plasma BDNF levels were decreased in humans with T2DM and were independent of obesity in a study by Krabbe et al. [13]. In the same study, plasma BDNF levels were inversely associated with fasting plasma glucose. There are conflicting data about BDNF and glucose metabolism association. This situation may be the result of ethnic differences.

In a previous study, BDNF heterozygous knockout (BDNF +/−) mice showed obesity and insulin resistance [21]. It is possible that the increase recorded in BDNF in the current study of T2DM may compensate for hyperinsulinemia and insulin resistance. Suwa et al. reported a relationship between fasting blood glucose, triglycerides, and HOMA-IR and serum BDNF values [15]. A previous study found that serum BDNF was associated with fasting insulin and HOMA-IR in T2DM [12]. Krabbe et al. reported that plasma BDNF was inversely associated with HOMA-IR but not insulin [13]. Our study demonstrated that serum BDNF levels were significantly positively correlated with HOMA-IR and triglyceride. Other studies showed that BDNF improved hepatic insulin resistance in diabetic animals [22] and that BDNF was positively correlated with triglyceride, total cholesterol, and LDL-C in humans [23]. BDNF contributes the same mechanism with leptin in regulating lipid metabolism [18]. Some authors observed that BDNF treatment of obese and diabetic animals has a positive effect on glucose and lipid metabolism, with one study demonstrating that subcutaneous administration of BDNF reduces food intake and ameliorates impaired glucose tolerance in diet-induced obese mice [9]. BDNF also reduces serum insulin and glucose levels when injected into diabetic rats [24]. The results of these studies show that BDNF may have a role in the treatment of diabetes and dyslipidemia.

Previous studies described an association of plasma BDNF with inflammatory conditions [25, 26]. Some studies demonstrated increased BDNF expression in inflamed bladder tissue and in airway epithelium during allergic airway inflammation [27, 28]. Shin et al. showed that the plasma BDNF level was positively correlated with inflammatory cytokines in hemodialysis patients, suggesting that plasma BDNF might reflect uremic inflammation in patients undergoing hemodialysis [29]. In our study, serum BDNF levels showed a positive correlation with WBC but not CRP levels. Inversely, a previous study reported a correlation between BDNF and CRP in T2DM patients [13]. The increase in BDNF levels may be associated with protecting neurons from inflammatory injury [30].

In the logistic regression analysis, BDNF was independently associated with T2DM, irrespective of age, BMI, and CRP. Patients who had a serum BDNF level more than 137 pg/mL were a predictive value like HbA1c for the diabetes diagnosis with a sensitivity of 71.79% and a specificity of 68%. It had a positive predictive value of 87.5% and a negative predictive value of 43.59%. This novel research suggests that serum BDNF may be used as a prediction data for T2DM like HgA1c in future. More importantly, the standardization and cut-off values of serum BDNF had been controversial in previous studies. For the first time, current study suggests a cut-off point for serum BDNF of type 2 diabetes mellitus.

There are some limitations in this study. First, this was a cross-sectional study of a relatively small number of patients. A larger patient population should be recruited. Second, we measured levels of BDNF but not those of other neurotrophins. Third, we measured levels of WBCs and CRP to determine the association between BDNF values and inflammation but not those of other serum inflammatory markers, such as interleukin-6 or tumor necrosis factor.

In conclusion, serum BDNF level was higher in patients with T2DM, and the cut-off predictive value of BDNF was 137 pg/mL. The findings of this study suggest that BDNF may contribute to glucose and lipid metabolism and inflammation.

## Figures and Tables

**Table 1:** Clinical characteristics of patients and controls.

	Patient group (*n* = 88)		Control group (*n* = 33)	
	Mean ± s.d./*n*, %		Mean ± s.d./*n*, %	
Age (years)	60.03	±12.22	58.82	±10.80
Gender				
Male	38	43.2%	17	51.5%
Female	50	56.8%	16	48.5%
BMI (kg/m^2^)	31.52	±5.80	26.87	±3.89^***^
Waist circumference (cm)	108.75	±14.07	94.36	±9.75^***^
Systolic pressure (mmHg)	130.51	±14.40	118.03	±8.00^***^
Diastolic pressure (mmHg)	81.25	±8.17	74.24	±7.82^***^
BDNF (pg/mL)	206.81	±107.32	130.84	±59.81^***^
Fasting blood glucose (mg/dL)	170.36	±91.21	85.33	±11.49^***^
Fasting insulin (*µ*U/mL)	9.26	±4.92	5.48	±2.13^***^
HOMA-IR	3.73	±3.05	1.16	±0.48^***^
HbA1c (%)	8.30	±2.37	5.42	±0.52^***^
Triglyceride (mg/dL)	175.95	±108.67	156.30	±65.02
Total cholesterol (mg/dL)	212.21	±35.97	199.20	±52.50
LDL (mg/dL)	128.18	±32.17	115.52	±44.26
HDL (mg/dL)	47.01	±13.54	48.27	±13.20
CRP (mg/L)	7.84	±2.84	2.57	±1.07^***^
ESR (mm/hr)	26.09	±10.54	19.79	±5.58^***^
White blood cell (×10^3^/*µ*L)	7278.41	±2190.07	6624.24	±1683.38
Ferritin (ng/mL)	84.88	±38.69	44.52	±26.58
Creatinine clearance (mL/min)	91.74	±40.88	158.54	±83.23^***^
Oral antidiabetic drug	56	64%	0	0%
Oral antidiabetic drug plus insulin	16	18%	0	0%
Insulin	16	18%	0	0%
Antihypertension	54	61%	0	0%
Antilipid	26	30%	0	0%
Smoking history	31	35.2%	0	0%

Student's *t*-test, Yates' continuity correction test, and Mann-Whitney *U* test.

Statistical significance: ^***^ *P* < 0.001.

BMI: body mass index, BDNF: brain-derived neurotrophic factor, HOMA-IR: homeostasis model assessment of insulin resistance, LDL: low-density lipoprotein, HDL: high-density lipoprotein, CRP: C-reactive protein, and ESR: erythrocyte sedimentation rate.

**Table 2:** Relationship between HOMA-IR, triglyceride levels, WBC levels, and BDNF in T2DM patients.

	Patient group	
	*r*	*P*
BDNF and HOMA-IR	0.281	**0.021** ^*^
BDNF and triglyceride	0.265	**0.019** ^*^
BDNF and WBC	0.355	**0.001** ^***^

Statistical significance: ^*^ *P* < 0.05, ^***^ *P* < 0.001.

BDNF: brain-derived neurotrophic factor, HOMA-IR: homeostasis model assessment of insulin resistance, and WBC: white blood cell.

**Table 3:** Logistic regression analysis of independent factors associated with T2DM.

	*P*	Exp. (*B*) Odds	95% CI for Exp. (*B*)	
			Lower	Upper
Age	**0.013** ^*^	1.093	1.019	1.172
CRP	**0.014** ^*^	1.806	1.129	2.888
BDNF	**0.001** ^***^	9.987	2.417	41.267
BMI	**0.045** ^*^	7.377	1.043	52.197

Statistical significance: ^*^ *P* < 0.05, ^***^P < 0.001.

CRP: C-reactive protein, BDNF: brain-derived neurotrophic factor, and BMI: body mass index.

**Table 4:** Diagnostic screening tests results for BDNF.

BDNF	Sensitivity	Specificity	Positive predictive value	Negative predictive value	Validity
≥120	76.92	60.00	85.71	45.45	72.82
≥128	74.36	60.00	85.29	42.86	70.87
≥130	73.08	60.00	85.07	41.67	69.90
≥137	**71.79**	**68.00**	**87.50**	**43.59**	**70.87**
≥142	69.23	68.00	87.10	41.46	68.93
≥145	66.67	72.00	88.14	40.91	67.96
≥150	65.38	72.00	87.93	40.00	66.99
